# Functional In Vitro Model of the Canine Corpus Luteum: Isolation, Culture and Characterization of Steroidogenically Active Luteal Cells

**DOI:** 10.3390/biomedicines14071444

**Published:** 2026-06-25

**Authors:** Patrycja Kalak, Paulina Bugno, Jan P. Madej, Mateusz Speruda, Antoni Szumny, Maciej Janeczek, Wojciech Niżański, Tomasz Gębarowski, Michał Dzięcioł

**Affiliations:** 1 Department of Reproduction and Farm Animals, Wrocław University of Environmental and Life Sciences, 50-375 Wrocław, Poland; wojciech.nizanski@upwr.edu.pl (W.N.); michal.dzieciol@upwr.edu.pl (M.D.); 2 Department of Biostructure and Animal Physiology, Wrocław University of Environmental and Life Sciences, 51-631 Wrocław, Poland; paulina.bugno@upwr.edu.pl (P.B.); maciej.janeczek@upwr.edu.pl (M.J.); 3 Department of Immunology, Pathophysiology and Veterinary Preventive Medicine, Wrocław University of Environmental and Life Sciences, 50-375 Wrocław, Poland; jan.madej@upwr.edu.pl; 4 Department of General and Experimental Pathology, Wrocław Medical University, 50-368 Wrocław, Poland; mateusz.speruda@umw.edu.pl; 5 Department of Food Chemistry and Biocatalysis, Wrocław University of Environmental and Life Sciences, 50-357 Wrocław, Poland; antoni.szumny@upwr.edu.pl

**Keywords:** canine corpus luteum, primary luteal cell culture, canine ovary, cell isolation, progesterone secretion, steroidogenesis, cryobanking, reproductive physiology

## Abstract

**Background/Objectives:** The corpus luteum (CL) in the dog is the sole source of progesterone (P4) during diestrus and pregnancy, making it a key regulator of reproductive function. However, robust and functionally validated in vitro models of canine luteal cells remain limited. This study aimed to establish and characterize a reproducible primary culture system of canine luteal cells with preserved steroidogenic activity and regulatory responsiveness. **Methods:** Ovaries containing CLs were collected from five clinically healthy bitches undergoing routine ovariohysterectomy (OHE). Luteal tissue was mechanically fragmented and enzymatically digested using collagenase type II. Primary cultures were established using an explant-based approach and maintained in Dulbecco’s Modified Eagle Medium/Ham’s F-12 nutrient mixture (DMEM/F12) or Roswell Park Memorial Institute medium 1640 (RPMI 1640) supplemented with 20% fetal bovine serum (FBS). Cellular morphology, proliferation, expression of steroidogenic markers—steroidogenic acute regulatory protein (STAR) and 3β-hydroxysteroid dehydrogenase type 1 (HSD3B1), P4 secretion, and responsiveness to forskolin stimulation were evaluated. **Results:** Cultured luteal cells exhibited stable attachment, proliferation, and a predominantly spindle-shaped morphology. Both media supported maintenance of a steroidogenic phenotype, while RPMI 1640 enabled enhanced proliferation, allowing expansion up to passage three and efficient cryobanking. Cells remained functionally active, secreting progesterone for up to 28 days in vitro. Forskolin stimulation increased progesterone secretion up to 2.7-fold, confirming preserved cyclic AMP-dependent steroidogenic responsiveness. **Conclusions:** The canine CL is a reliable source of functionally competent luteal cells, and the established culture system represents a physiologically relevant in vitro model. To our knowledge, this is the first functionally validated in vitro model of the canine CL. This platform enables controlled investigations of luteal function, endocrine regulation, and mechanisms of P4 synthesis, supporting its application in mechanistic and translational reproductive research.

## 1. Introduction

Corpus luteum (CL) is a transient endocrine gland that forms in the ovary after ovulation as a result of luteinization of granulosa and theca cells within the preovulatory follicle [1,2]. Following follicular rupture, the newly formed CL occupies the site of the ovulated follicle within the ovarian cortex, where it develops through the stages of early (developing), mature (functional), and late (regressing) luteal phases. This structure plays a central role in the reproductive cycle and maintenance of pregnancy by secreting progesterone (P4)—a hormone essential for preparing the endometrium for implantation, sustaining pregnancy, and supporting normal embryonic and fetal development [2,3]. Adequate luteal P4 secretion is therefore critical for reproductive success, while insufficient production commonly results in embryonic or fetal loss across mammalian species [1,2].

CL is characterized by an extensive vascular network and a dynamic cellular composition that includes steroidogenic, endothelial, pericytic, fibroblastic, and immune cell populations [4,5,6,7,8]. While much of the early research focused on the steroidogenic cell population due to its pivotal role in P4 biosynthesis [5,9,10], subsequent research has emphasized the importance of non-steroidogenic cells that modulate the luteal microenvironment, paracrine signaling, and angiogenic processes [5,11,12,13]. Luteal cells are commonly classified into large (LLC) and small (SLC) luteal cells, which differ in origin, morphology, and endocrine activity [6,14,15,16]. In general, LLCs originate primarily from granulosa cells, whereas SLCs arise from theca interna cells [17,18,19,20,21], as shown in ruminants and other mammals, although some species-specific differences exist [8,22]. In addition to steroidogenic cells, the CL contains a variety of non-steroidogenic populations that contribute to extracellular matrix remodeling, angiogenesis, and immune regulation within the luteal microenvironment [23,24].

In the domestic dog (*Canis familiaris*), CL displays several distinctive features compared with other species. Its functional activity persists for several weeks regardless of whether pregnancy occurs, and importantly, the canine placenta lacks steroidogenic capacity. Consequently, CL serves as the sole source of P4 during both pregnancy and the non-pregnant luteal phase [25,26]. This makes luteal function critical for the progression of the estrous cycle, and any dysfunction may result in infertility or early embryonic loss. These unique characteristics make canine CL a particularly interesting model for studying endocrine regulation, luteal maintenance, and regression [27].

Mammalian cell culture systems have become indispensable tools in reproductive and biomedical research, offering controlled conditions for dissecting cellular and molecular mechanisms underlying luteal physiology [28,29]. Primary luteal cell cultures have been successfully established in various species and applied to study distinct regulatory pathways and endocrine functions.

In primates, including humans and non-human primates, luteal cell isolates have been obtained from surgically collected ovarian tissue, most often during gynecological procedures, reproductive surgeries, or scheduled necropsies in research colonies. Isolation protocols typically involve mechanical fragmentation of luteal tissue followed by enzymatic digestion to release mixed luteal cell populations. These in vitro luteal models have been extensively used to investigate steroidogenic pathways, luteotropic and luteolytic signaling, angiogenesis within the CL, and the endocrine regulation of the menstrual cycle. Importantly, primate luteal cell cultures have provided key insights into the mechanisms underlying luteal insufficiency and early pregnancy loss, with direct relevance to human reproductive medicine and assisted reproductive technologies [30,31].

In livestock species—including cattle, pigs, sheep, and goats—luteal cell isolation protocols have typically relied on obtaining CLs from slaughterhouse-derived ovaries, followed by mechanical mincing and enzymatic digestion, most commonly using collagenase (often supplemented with DNase). In many studies, particularly those focused on ruminants, density gradient centrifugation (e.g., Percoll or Ficoll) was employed to enrich or separate distinct luteal cell fractions, including large and small steroidogenic cells [14,32,33]. Earlier work in cattle and pigs primarily described mixed luteal cell preparations without subpopulation fractionation but laid the foundation for the development of in vitro luteal models [34,35]. These culture systems have been extensively used to elucidate the hormonal regulation of the estrous cycle, prostaglandin-mediated luteolysis, cytokine signaling, and mechanisms underlying luteal angiogenesis and regression. Importantly, luteal cell cultures in livestock species have also played a significant role in applied reproductive research, contributing to the optimization of fertility management, improvement of reproductive efficiency, and the development of effective breeding strategies in domestic animals.

In small animal species, luteal cell isolation has been performed in rodents, felids, and canids using broadly comparable enzymatic approaches, albeit with species-specific modifications, primarily to investigate fundamental mechanisms of luteal steroidogenesis, cell differentiation, angiogenesis, and apoptotic pathways under controlled experimental conditions. In rodents, corpora lutea collected at defined stages of pregnancy or the estrous cycle were typically minced and subjected to collagenase digestion—occasionally supplemented with DNase or hyaluronidase—followed by filtration and centrifugation to obtain mixed or fractionated luteal cell populations. Such protocols, as employed in rats and mice, enabled detailed studies of luteal cell subpopulations, steroidogenic activity, and apoptosis-related pathways [36,37]. In cats, luteal cell isolation was performed using protocols adapted from livestock species, most often involving collagenase digestion, sequential filtration, and low-speed centrifugation, providing a basis for examining progesterone synthesis and regulatory factors influencing feline luteal function [38]. In dogs, CLs obtained predominantly during elective ovariohysterectomy (OHE) were isolated through collagenase-based enzymatic dissociation, sometimes supplemented with DNase or additional purification steps, yielding short-term cultures used to investigate P4 production, steroidogenic enzyme expression, and early luteal differentiation events [39].

Nevertheless, despite these advances, studies on canine luteal cell isolation remain scarce, and no reproducible and functionally validated in vitro model of the canine corpus luteum has been established to date. Furthermore, no primary or long-term canine luteal cell lines are currently available, which limits functional and pharmacological studies of luteal physiology in this species. This gap is particularly significant given the unique endocrine profile of the domestic dog, in which the corpus luteum constitutes the sole source of progesterone and remains functionally active for approximately two months after ovulation, regardless of pregnancy status. These species-specific features underscore the need for a reliable canine model for studying endocrine regulation, luteal maintenance, and progesterone-dependent reproductive processes.

Therefore, the aim of the present study was to establish and functionally characterize a reproducible primary in vitro model of the canine corpus luteum, with particular emphasis on steroidogenic activity and regulatory responsiveness. Such a model provides a valuable platform for mechanistic studies of luteal function, luteolysis, and hormonal signaling under controlled conditions.

## 2. Materials and Methods

### 2.1. Tissue Collection

Ovaries containing CLs were obtained from non-pregnant bitches in the luteal phase of the estrous cycle during routine OHE procedures performed at the Department of Reproduction with the Clinic of Farm Animals, Wrocław University of Environmental and Life Sciences, Poland. The animals were privately owned and underwent elective OHE performed for non-experimental, prophylactic clinical reasons unrelated to the present study. Because the biological material originated from medical waste generated during routine veterinary procedures, the study did not require approval from the Local Ethical Committee for Animal Experimentation. Written informed consent for the use of excised tissues for research purposes was obtained from all owners prior to surgery.

Immediately after surgical removal, ovaries containing CLs were immersed in sterile phosphate-buffered saline (PBS; pH 7.4) supplemented with gentamicin (50 µg/mL) and transported to the Cell Culture and Advanced Therapy Laboratory, Department of Biostructure and Animal Physiology, Wrocław University of Environmental and Life Sciences, for further processing.

From each ovary, representative tissue fragments were excised and allocated for histological examination to confirm ovulation and the presence of CLs. The remaining ovarian tissue was used for subsequent isolation of luteal structures intended for primary cell culture experiments.

### 2.2. Determination of Clinical Health Status and Estrous Cycle Stage

All donor bitches were considered clinically healthy at the time of surgery based on a standard physical examination performed by a licensed veterinarian. In four out of five animals, routine preoperative blood examinations were additionally conducted, including complete blood count and serum biochemical analysis. All hematological, biochemical, and hormonal analyses were performed using peripheral blood collected during a single blood sampling procedure. Hematological parameters were assessed using an automated analyzer (IDEXX ProCyte Dx^®^, IDEXX Laboratories, Westbrook, ME, USA), while serum biochemistry was evaluated with an automated chemistry analyzer (IDEXX Catalyst One^®^, IDEXX Laboratories, Westbrook, ME, USA).

Reproductive status was assessed based on clinical signs of estrus, followed by post-estrus determination of serum P4 concentrations to confirm the luteal phase of the estrous cycle. P4 concentrations were measured in peripheral blood using an enzyme-linked fluorescent assay (ELFA; miniVIDAS^®^, bioMérieux, Craponne, France). According to Brugger et al. (2011) [40], the assay demonstrates low intra- and inter-assay variability, with coefficients of variation generally below 5% across clinically relevant P4 concentrations [40]. Blood sampling and P4 measurements were consistently performed during the morning hours to minimize the potential influence of circadian variation in P4 secretion and to allow more reliable comparison of hormonal dynamics [41].

### 2.3. Histological Analysis of the Corpora Lutea

Tissue samples designated for histology were fixed in 10% buffered formalin (Sigma-Aldrich, St. Louis, MO, USA) for 48 h and processed according to standard protocols for formalin-fixed paraffin-embedded (FFPE) preparation. Briefly, samples were dehydrated in a graded ethanol series, cleared twice in xylene, and embedded in paraffin.

From FFPE blocks, 5-µm-thick sections were cut using a microtome and subjected to standard deparaffinization procedures. Sections were stained with hematoxylin and eosin (H&E) using Delafield hematoxylin (Roth GmbH, Karlsruhe, Germany) and eosin (POCH S.A., Gliwice, Poland).

Histological slides were examined and documented under a light microscope (Nikon Eclipse Ni; Nikon, Melville, NY, USA) equipped with a digital camera using NIS-Elements BR version 5.41 imaging software (Nikon Corporation, Tokyo, Japan) to verify the presence and morphology of CLs.

### 2.4. Isolation of Luteal Tissue for Cell Culture

Following tissue allocation for histological analysis described above, the remaining ovarian tissue containing CLs was processed under sterile conditions in a laminar flow hood.

Prior to cell isolation, tissue culture flasks were prepared using MSC Attachment Solution (Biological Industries, Kibbutz Beit-Haemek, Israel) according to the manufacturer’s instructions. Briefly, the culture surface was covered with MSC Attachment Solution and incubated at room temperature for 30 min. After incubation, excess solution was removed immediately before cell seeding without additional washing.

CLs were dissected from the ovarian tissue and transferred to sterile Petri dishes containing PBS supplemented with gentamicin (Sigma-Aldrich, St. Louis, MO, USA). The isolated structures were rinsed several times to remove blood residues and mechanically fragmented into small pieces (approximately 1–2 mm^3^) using sterile scissors. The minced tissue fragments were transferred into digestion medium containing collagenase type II (Gibco/Thermo Fisher Scientific, Waltham, MA, USA) at a final concentration of 1.5 mg/mL prepared in the respective basal culture medium. Two basal media were evaluated: Roswell Park Memorial Institute 1640 medium (RPMI 1640; Biological Industries, Kibbutz Beit-Haemek, Israel) and Dulbecco’s Modified Eagle Medium/Ham’s F-12 nutrient mixture (DMEM/F12; Biological Industries, Kibbutz Beit-Haemek, Israel), both supplemented with gentamicin (50 µg/mL). Enzymatic digestion was carried out for 2 h at 37 °C under gentle agitation. During incubation, the suspension was periodically mixed to facilitate tissue dissociation.

**Figure 1:**
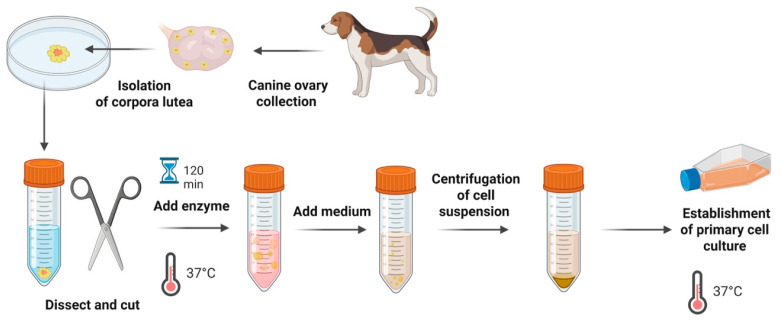
Workflow of canine luteal cell isolation and primary culture establishment. Canine ovaries were collected during routine ovariohysterectomy (OHE) procedures, and corpora lutea (CLs) were isolated from ovarian tissue. The tissue was mechanically fragmented and enzymatically digested with collagenase (120 min, 37 °C). After the addition of culture medium and centrifugation, the resulting cell suspension containing dispersed cells and tissue explants was seeded into culture flasks and maintained under standard culture conditions (37 °C, 5% CO_2_) to establish primary luteal cell cultures. Created in BioRender. Gębarowski, T. (2026) https://BioRender.com/9a24t5c, accessed on 22 June 2026.

After digestion, the cell suspension was gently dissociated by repeated pipetting and centrifuged at 300× *g* for 5 min at room temperature. The supernatant was discarded, and the cell pellet was resuspended in complete culture medium. The complete culture medium consisted of RPMI 1640 or DMEM/F12 supplemented with 20% fetal bovine serum (FBS; Biological Industries, Kibbutz Beit-Haemek, Israel) and gentamicin (50 µg/mL). The cell suspension was seeded into MSC Attachment-coated culture flasks and incubated at 37 °C in a humidified atmosphere containing 5% CO_2_. The first medium change was performed after 24 h to remove non-adherent cells and tissue debris. Subsequently, the culture medium was replaced every 2–3 days (Figure 1).

### 2.5. Culture and Passaging of Primary Canine Luteal Cells

After initial isolation and seeding onto MSC Attachment-coated culture flasks, primary canine luteal cells were maintained under standard culture conditions. The culture medium was replaced as required according to the rate of cell growth. Cell proliferation and morphology were monitored regularly using an inverted phase-contrast microscope (EVOS FL, Thermo Fisher Scientific, Waltham, MA, USA).

Cells were passaged when cultures reached approximately 80% confluence. For passaging, the culture medium was removed, and the cells were rinsed with sterile PBS to eliminate residual serum. Cells were detached using standard enzymatic dissociation solution (TrypLe Ex, Thermo Fisher Scientific, Waltham, MA, USA) and incubated at 37 °C until cell detachment was observed microscopically. The enzymatic reaction was stopped by the addition of complete culture medium. The cell suspension was then collected and centrifuged at 300× *g* for 5 min, and the resulting cell pellet was resuspended in fresh complete medium. After the first passage, cells were seeded into standard tissue culture flasks without MSC Attachment coating and maintained under the same culture conditions as primary cultures.

Throughout the culture period, special attention was paid to the detection of potential microbial contamination. Cultures were routinely inspected during microscopic examination for the presence of abnormal particles, changes in cell morphology, or signs of bacterial or fungal growth. In addition, the macroscopic appearance of the culture medium was monitored, and turbidity or color changes inconsistent with normal culture conditions were considered indicative of possible contamination.

### 2.6. Workflow of Primary Canine Luteal Cell Culture and Expansion

Primary canine luteal cells were expanded according to a standardized culture workflow from initial isolation to cryopreservation.

Following enzymatic isolation, cells and tissue explants were seeded into MSC Attachment-coated T25 culture flasks (passage 0, P0) and maintained in complete culture medium consisting of RPMI 1640 or DMEM/F12 supplemented with 20% FBS and gentamicin (50 µg/mL). Cultures were maintained under standard incubation conditions, and the culture medium was replaced twice per week.

After reaching approximately 70–80% confluence, primary cultures were passaged using trypsin–EDTA (TrypLe Ex, Thermo Fisher Scientific, Waltham, MA, USA) and transferred into T75 culture flasks (passage 1, P1). From this stage onward, cultures were maintained in their respective basal media without switching between DMEM/F12 and RPMI 1640.

For RPMI cultures, cells were further expanded by passaging from a single T75 flask into two T75 flasks (passage 2, P2). Cryopreservation material was obtained from one or both T75 flasks depending on cell yield. When sufficient cell numbers were obtained, cultures were expanded to the third passage (P3), and cryopreservation material was again collected from T75 flasks.

For DMEM/F12 cultures, expansion after passaging was limited; therefore, cryopreservation was typically performed at early passage (P1) using cells obtained from T75 flasks.

**Figure 2:**
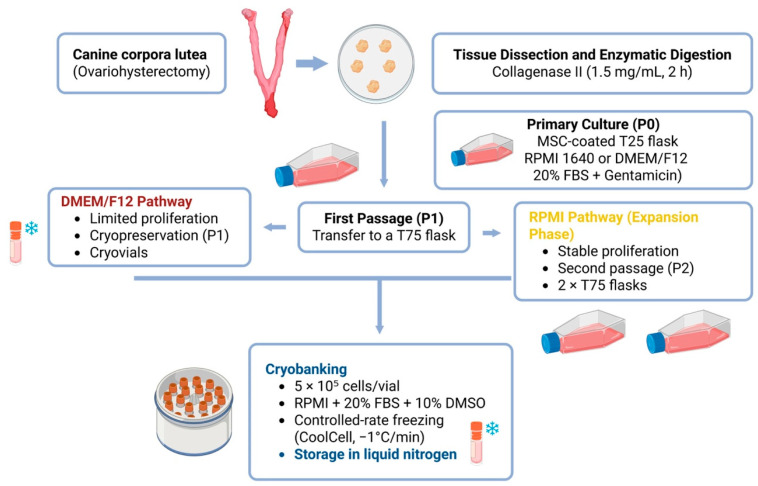
Workflow of primary canine luteal cell isolation, expansion, and cryopreservation. Primary canine luteal cells were isolated from CLs and cultured in MSC Attachment-coated T25 flasks (P0). After P1, cells were expanded in T75 flasks. Roswell Park Memorial Institute medium 1640 (RPMI) cultures were further expanded to two T75 flasks (P2) and subsequently to P3 prior to cryopreservation, whereas Dulbecco’s Modified Eagle Medium/Ham’s F-12 nutrient mixture (DMEM/F12) cultures were typically cryopreserved at P1 due to limited proliferative capacity. Created in BioRender. Gębarowski, T. (2026) https://BioRender.com/edkkmqb, accessed on 22 June 2026.

This standardized workflow allowed reproducible expansion and cryobanking of primary canine luteal cells from all donors (Figure 2).

### 2.7. Cell Counting and Viability Assessment

Cell yield and viability were assessed immediately after isolation and, when required, after passaging. Cell concentration and viability were determined using an NC-200™ automated cell counter (ChemoMetec, Allerød, Denmark) based on fluorescent dye exclusion according to the manufacturer’s instructions.

Briefly, an aliquot of the cell suspension was mixed with Via1-Cassette™ reagents (ChemoMetec, Allerød, Denmark) and loaded into Via1-Cassette™ slides. Measurements were performed using the predefined protocol for mammalian cells, and the instrument automatically provided total cell concentration, viable cell concentration, and percentage of viable cells. The obtained values were used to standardize seeding density for primary cultures and subsequent experiments.

### 2.8. Evaluation of Culture Expansion and Population Doubling Time

To compare the expansion characteristics of primary canine luteal cells maintained in RPMI 1640 and DMEM/F12 media, cell numbers were determined at each passage using an NC-200™ automated cell counter (ChemoMetec, Allerød, Denmark). Cell counts obtained immediately before passaging were used to calculate culture expansion parameters.

The fold expansion ratio was calculated as the ratio of harvested cells to the number of cells initially seeded for a given passage:

Fold expansion = harvested cell number/seeded cell number

In addition, the population doubling time (DT) was estimated according to the following equation:

DT = t × log(2)/log(N_t_/N_0_)

where:

- t = culture time (hours),
- N_0_ = number of seeded cells
- N_t_ = number of harvested cells at the time of passaging.

The time required to reach passaging confluence and the calculated doubling times were used as quantitative indicators of culture expansion efficiency under the respective culture conditions.

### 2.9. Immunofluorescence Staining of Primary Canine Luteal Cells 

Primary canine luteal cells were characterized by immunofluorescence staining for steroidogenic markers. Immunofluorescence analyses were performed using post-passage cultures. Cells maintained in DMEM/F12 medium were evaluated after P1, whereas RPMI 1640 cultures were evaluated at P2 and P3. Cells were seeded onto 10-well chamber slides (Greiner Bio-One, Kremsmünster, Austria) and maintained under standard culture conditions until approximately 70% confluence was reached.

Cells were fixed with 4% paraformaldehyde for 15 min at room temperature and subsequently rinsed with PBS containing 0.1% Tween-20 (PBST). Cell membranes were permeabilized by incubation with 0.1% Triton X-100 (Sigma-Aldrich, St. Louis, MO, USA) for 10 min, followed by washing with PBST. Non-specific antibody binding sites were blocked by incubation in PBST containing 1% bovine serum albumin (BSA; Sigma-Aldrich, St. Louis, MO, USA) and 10% normal goat serum (NGS; Sigma-Aldrich, St. Louis, MO, USA) for 30 min at room temperature. After blocking, cells were rinsed with PBST. Cells were incubated for 1.5 h with primary antibodies against steroidogenic acute regulatory protein (STAR) and 3β-hydroxysteroid dehydrogenase type 1 (HSD3B1): rabbit polyclonal anti-STAR (1:100, AffBiotech, Cincinnati, OH, USA) and rabbit polyclonal anti-HSD3B1 (1:100, Biorbyt, Durham, UK). After washing with PBST, cells were incubated for 1.5 h with secondary antibodies: goat anti-rabbit IgG (H + L), fluorescein (Vector Laboratories, Newark, CA, USA) and Cy™5 AffiniPure^®^ donkey anti-rabbit IgG (H + L) (Jackson ImmunoResearch, Cambridgeshire, UK). After incubation with secondary antibodies, cells were washed with PBST and mounted using Vectashield mounting medium with 4′,6-diamidino-2-phenylindole (DAPI) (Vector Laboratories, Newark, CA, USA) for nuclear staining. Negative control slides were processed identically except that the primary antibody was omitted and replaced by antibody diluent. Cells were then incubated with the corresponding secondary antibody under the same conditions. No specific fluorescence signal was observed in the negative controls. Immunofluorescence staining was independently performed on cultures established from all five donor animals to confirm staining reproducibility. Fluorescence images were acquired using an EVOS FL fluorescence microscope (Thermo Fisher Scientific, Waltham, MA, USA) with a 20× objective lens. The following fluorescence filter cubes were used: DAPI (Ex 357–377 nm/Em 447–460 nm), GFP/FITC (Ex 470–490 nm/Em 510–540 nm), and Cy5 (Ex 620–640 nm/Em 660–700 nm). Scale bar = 200 µm.

### 2.10. Assessment of Progesterone Secretion by Primary Canine Luteal Cell Cultures

P4 secretion by primary canine luteal cell cultures was assessed under in vitro conditions. After reaching the appropriate confluence, culture media were collected for hormonal analysis at defined time points. Prior to sample collection, the culture medium was replaced with fresh complete medium to ensure that measured P4 concentrations reflected de novo P4 secretion by the cultured cells during the incubation period.

P4 concentrations in culture supernatants were determined using a competitive enzyme-linked immunosorbent assay (ELISA) kit (Progesterone ELISA Kit, Cayman Chemical, Ann Arbor, MI, USA, Item No. 582601). The assay is based on a competitive immunoassay principle and allows quantitative determination of P4 in cell culture media. According to the manufacturer, the assay has a working range of 7.8–1000 pg/mL and a sensitivity of approximately 10 pg/mL. Reported intra-assay and inter-assay coefficients of variation ranged from 4.9 to 12.1% and 1.5–16.4%, respectively. Samples and standards were analyzed in duplicate, and progesterone concentrations were calculated from a calibration curve generated using serial dilutions of the supplied standards and a four-parameter logistic (4PL) regression model.

To correct for non-specific binding, the mean absorbance value of the non-specific binding wells (NSB) was subtracted from all measured values. The corrected absorbance values were expressed as a fraction of the maximum binding signal (B_0_) according to the equation:

B/B_0_ = (A_sample_ − A_NSB_)/(A_B0_ − A_NSB_)

where:

- A_sample_ = absorbance of the analyzed sample
- A_NSBA_ = mean absorbance of NSB
- A_B0_ = mean absorbance of maximum binding wells.

P4 concentrations were obtained by interpolation of B/B_0_ values from the standard curve and expressed as pg/mL of culture medium. For clarity of presentation, selected results were additionally converted to ng/mL.

Cell-free culture medium incubated under identical conditions was analyzed as a background control for progesterone determination.

### 2.11. Cell Harvesting, Cryopreservation and Cryobanking

Primary canine luteal cells were harvested for cryopreservation at selected passages depending on their proliferative capacity in culture. Cells were maintained in either DMEM/F12 or RPMI 1640 medium, without switching between media within individual cultures. Parallel cultures in both media were established for donors 3–5, whereas cultures derived from donors 1 and 2 were maintained exclusively in DMEM/F12.

Prior to cryopreservation, cells were detached using trypsin–EDTA solution, collected by centrifugation at 300× *g* for 5 min, and resuspended in freezing medium. Cell number and viability were determined using an NC-200™ automated cell counter with Via1-Cassette™ slides according to the manufacturer’s instructions. Only cell suspensions with satisfactory viability were used for cryopreservation.

Cells were cryopreserved at a target density of approximately 5 × 10^5^ cells per cryovial (±20%). The freezing medium consisted of RPMI 1640 supplemented with 20% FBS and 10% dimethyl sulfoxide (DMSO; Sigma-Aldrich, St. Louis, MO, USA). Cryovials were placed into a controlled-rate freezing container (Corning^®^ CoolCell™ LX Cell Freezing Container, Corning Inc., Corning, NY, USA), providing a cooling rate of approximately −1 °C/min, and stored overnight at −80 °C. Subsequently, samples were transferred to long-term storage in liquid nitrogen.

### 2.12. Post-Thaw Viability and Growth Assessment

To verify the effectiveness of the cryopreservation procedure, selected cryovials were subjected to test thawing and subsequent culture. Cryovials were removed from liquid nitrogen storage and rapidly thawed by manual warming until only a small ice crystal remained in the vial.

Immediately after thawing, the cell suspension was transferred to a sterile tube containing pre-warmed complete culture medium to dilute the cryoprotectant. The cells were then centrifuged at 300× *g* for 5 min, and the pellet was resuspended in fresh complete medium.

Cells were seeded into tissue culture flasks and maintained under standard culture conditions (37 °C, 5% CO_2_). Post-thaw cell attachment, morphology, and proliferation were monitored by inverted phase-contrast microscopy during the following days of culture.

Successful recovery was defined as cell attachment to the culture surface and subsequent proliferation under standard culture conditions.

### 2.13. Mycoplasma Detection

To verify the microbiological quality of the established cultures, mycoplasma contamination screening was performed using the MycoStrip™ Mycoplasma Detection Kit (InvivoGen, San Diego, CA, USA) according to the manufacturer’s instructions. Culture supernatants collected from representative primary canine luteal cell cultures were analyzed at different stages of culture expansion. The assay is based on the detection of mycoplasma-associated enzymatic activity and provides a rapid qualitative assessment of mycoplasma contamination. Positive and negative controls supplied by the manufacturer were included in each assay. All tested cultures yielded negative results for mycoplasma contamination.

### 2.14. Forskolin Stimulation of Progesterone Secretion

To evaluate the steroidogenic responsiveness of primary canine luteal cells, P4 secretion was assessed following stimulation with forskolin [42,43,44]. Forskolin is a diterpene compound that directly activates adenylate cyclase, leading to an increase in intracellular cyclic adenosine monophosphate (cAMP) levels and stimulation of steroidogenesis in ovarian steroidogenic cells [45].

Forskolin (Sigma-Aldrich, St. Louis, MO, USA) was dissolved in DMSO to prepare a concentrated stock solution and diluted in complete culture medium immediately before use.

Cells from expanded post-passage cultures were maintained in complete medium until reaching approximately 70–80% confluence. Prior to stimulation, the culture medium was replaced with fresh complete medium. Forskolin was added to obtain final concentrations of 1 µM, 5 µM and 10 µM. Control cultures received an equivalent volume of DMSO without forskolin, and the final concentration of DMSO did not exceed 0.1% (*v*/*v*).

Cells were incubated under standard culture conditions (37 °C, 5% CO_2_) for 24 h, after which culture supernatants were collected and used for P4 determination by ELISA as described above.

### 2.15. Statistical Analysis

Statistical analyses were performed using GraphPad Prism 8.0.2 software (GraphPad Software Inc., San Diego, CA, USA). Data are presented as mean ± standard deviation (SD). Changes in progesterone secretion during long-term culture and the effects of forskolin stimulation were analyzed using the Friedman test for repeated measures. The Friedman test was selected due to the repeated-measures design and the relatively small sample size. Differences were considered statistically significant at *p* < 0.05.

For progesterone secretion experiments, analyses were performed using data obtained from five independent donor-derived primary luteal cell cultures (*n* = 5). For forskolin stimulation experiments, analyses were performed using four independent biological replicates (*n* = 4).

## 3. Results

### 3.1. Donor Characteristics and Clinical Status

The study included five (*n* = 5) privately owned bitches of various breeds, aged between 3 and 9 years (mean age: 5.4 years; median: 5 years). All donor animals were clinically healthy based on preoperative physical examination. When performed, preoperative hematological and biochemical blood tests revealed values within reference ranges or only minor deviations (e.g., slightly decreased MCV, MCHC, or reticulocyte counts), which were considered clinically insignificant by the attending veterinarian. Therefore, detailed blood test results are not presented.

In all donor animals, serum P4 concentrations exceeded 37 ng/mL, indicating the presence of a fully functional CL and confirming the mid-luteal (diestrus) phase of the estrous cycle at the time of tissue collection, as previously described in the domestic dog [25,46,47]. Although the exact day post-ovulation could not be determined for all animals, as the dogs included in the study were privately owned patients presented for routine OHE and not experimental animals, P4 concentrations were not monitored throughout the cycle, and blood samples were collected only once as part of the routine pre-surgical examination. Nevertheless, the consistently high circulating P4 concentrations observed were characteristic of the diestrus stage.

Detailed individual donor characteristics, including breed, age, onset of estrus based on clinical signs, serum P4 concentrations, dates of hormonal measurements and OHE, and the interval between P4 determination and tissue collection, are summarized in Supplementary Table S1.

### 3.2. Histological Characterization of Ovaries

**Figure 3:**
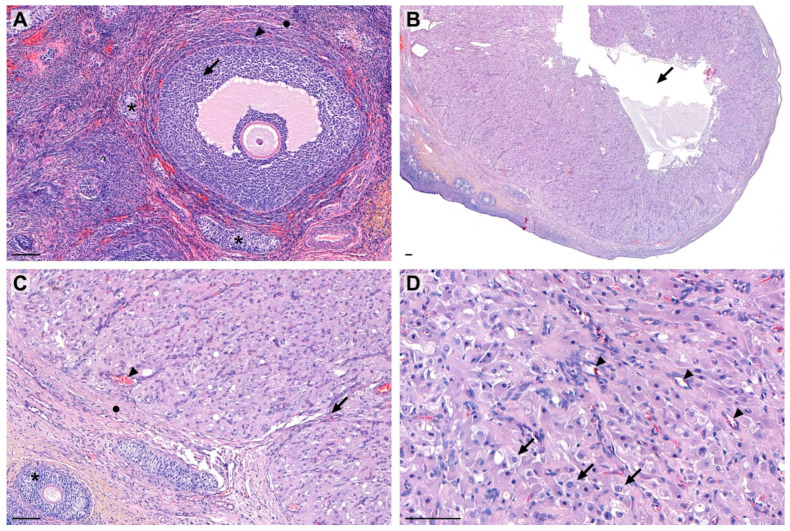
Histological micrographs of canine ovaries showing selected structures. (**A**). Tertiary (antral) follicle with granulosa cells (arrow), theca folliculi interna (arrowhead) and theca folliculi externa (dot); interstitial glands are also visible (asterisks) (**B**). CL is the largest structure within the ovary; the former antrum is still visible (arrow) (**C**). CL surrounded by tunica albuginea (dot), interspersed with bands of loose connective tissue (arrow) and supplied with blood vessels (arrowhead); the secondary follicle is also visible (asterisk) (**D**). high magnification of CL filled with luteal cells (arrows) and numerous blood vessels (arrowheads); H&E staining; scale bar—100 μm.

The material for cell culture was collected from the CLs, a component of the ovaries. Only ovaries with structures typical of female dogs and showing no histopathological changes were included in the study. Within the ovarian cortex (zona parenchymatosa), ovarian follicles of varying degrees of maturity, CLs, atretic follicles, and interstitial glands were observed (Figure 3). At this stage, fully developed CLs were the largest structures within the ovary. Each CL was surrounded by tunica albuginea and interspersed with bands of connective tissue. It was filled with numerous luteal cells and supplied by a dense network of blood vessels. The luteal cells were characterized by a large diameter (ca. 25–35 µm), polyhedral shape and large spherical or ovoid nucleus with a prominent nucleolus. In the cytoplasm, small lipid droplets were also observed. In the central area of some CL, the former antrum was still present.

### 3.3. Isolation Efficiency and Primary Culture Establishment

Primary cultures of canine luteal cells were established using an explant-based attachment approach following enzymatic digestion.

In MSC Attachment-coated culture flasks, luteal tissue fragments consistently adhered to the culture surface regardless of the basal medium used. Attached explants were typically observed within the first 24–48 h of culture. Subsequently, cells began to migrate from the attached tissue fragments and spread onto the culture surface, forming expanding areas of adherent cells during the following days of culture.

In the initial isolations (donors 1 and 2), both MSC Attachment-coated and uncoated culture flasks were used in parallel. In coated flasks containing DMEM/F12 medium, luteal tissue fragments attached efficiently and gave rise to migrating cell populations that developed into stable primary cultures. In contrast, in uncoated flasks only occasional single adherent cells were observed, and no consistent migration of cells from tissue explants occurred.

Based on these initial observations, all subsequent isolations (donors 3–5) were performed exclusively using MSC Attachment-coated culture flasks. Under these conditions, tissue explants consistently attached and reproducible primary cultures were obtained in both DMEM/F12 and RPMI 1640 media.

Although both basal media supported primary culture establishment, RPMI 1640 medium generally supported more stable cell growth and more consistent expansion of primary cultures compared with DMEM/F12 medium. A detailed comparison of growth characteristics in different media is presented in the following sections.

Details of isolation attempts and culture establishment efficiency for individual donors are summarized in Table 1.

### 3.4. Morphology and Growth Characteristics of Primary Canine Luteal Cells

**Figure 4:**
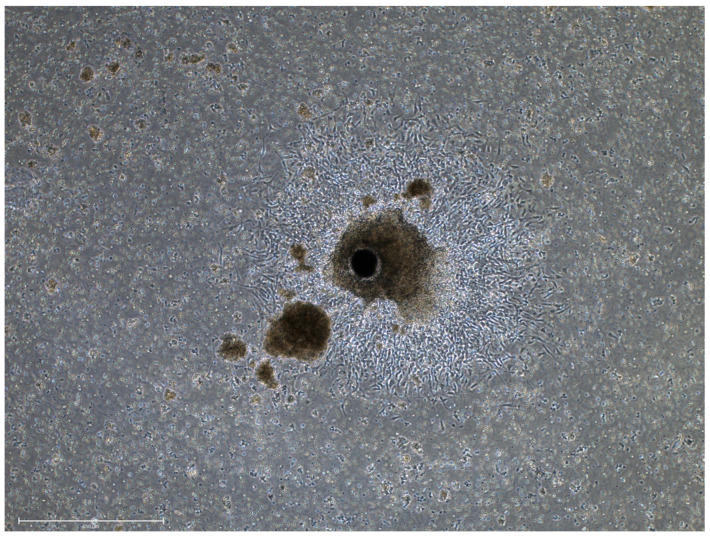
Representative microphotograph of primary canine luteal cell culture showing migrating cells originating from attached tissue explants during primary culture establishment. The image is representative of observations obtained in cultures derived from all donor animals. Adherent cells with developing spindle-shaped morphology are visible. Image acquired using an inverted phase-contrast microscope with a 4× objective lens.

**Figure 5:**
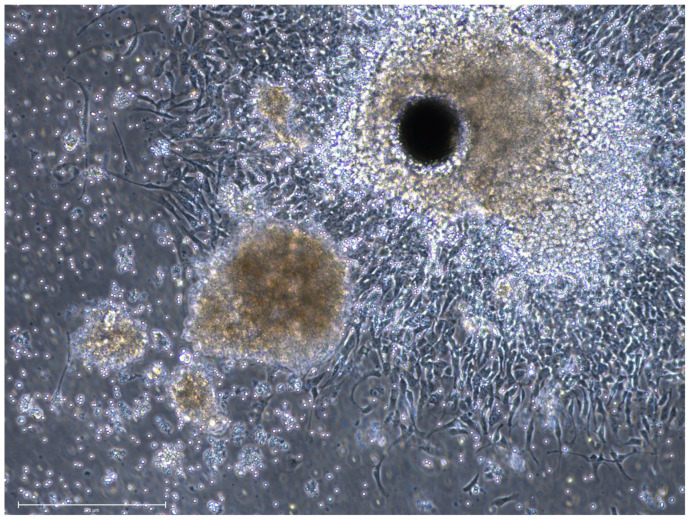
Representative microphotograph of primary canine luteal cells isolated from canine ovarian tissue and maintained under in vitro culture conditions. The image is representative of cultures derived from all donor animals. Adherent cells migrating from tissue explants are visible, forming dispersed cell populations on the culture surface. The cells exhibit predominantly elongated spindle-shaped morphology characteristic of adherent primary cultures. The image was acquired using an inverted phase-contrast microscope with a 10× objective lens.

Primary canine luteal cells formed adherent cultures originating predominantly from cells migrating from attached luteal tissue explants. Representative microscopic images illustrating explant attachment, cell migration, and subsequent development of primary cultures are shown in Figure 4 and Figure 5.

#### 3.4.1. Morphology and Growth Before the First Passage

During the initial phase of culture, luteal tissue explants adhered firmly to MSC Attachment-coated culture surfaces. Within the first days after seeding, cells migrating from the explants were observed in the immediate vicinity of attached tissue fragments. These cells initially displayed a rounded or oval morphology and were loosely attached to the culture surface. Over time, the cells gradually spread and developed a flattened morphology with increasing attachment strength.

In addition to cells migrating from tissue explants, a proportion of isolated single cells attached directly to the culture surface and contributed to the developing primary cultures. These cells typically appeared scattered between explants and showed progressive spreading during subsequent days of culture.

As culture progressed, the majority of adherent cells acquired an elongated spindle-shaped morphology characteristic of fibroblast-like adherent cells. Expanding clusters of proliferating cells formed around the explants and gradually developed into visible colonies. These colonies increased in size and eventually began to merge, forming areas of continuous cell growth.

The time required to reach subconfluence suitable for P1 differed depending on the basal medium used. Cultures maintained in RPMI 1640 medium typically reached approximately 70–80% confluence within about 7 days. In contrast, cultures maintained in DMEM/F12 medium required approximately 12–14 days to reach comparable confluence.

At the time of P1, most cells exhibited a uniform spindle-shaped morphology and formed a subconfluent adherent monolayer. In addition to spindle-shaped cells, a smaller subpopulation of cells representing approximately 10–20% of the culture displayed a polygonal or epithelial-like morphology characterized by a flattened and more regular, square, or cuboidal cell shape.

#### 3.4.2. Morphology and Growth After Passaging

Following enzymatic detachment, cells temporarily adopted a rounded morphology but reattached efficiently within approximately 12–24 h after reseeding. After reattachment, cells progressively regained their characteristic spindle-shaped morphology and resumed proliferation.

Clear differences in post-passaging growth behavior were observed between the two basal media. Cells cultured in RPMI 1640 medium continued to proliferate after P1 and maintained stable growth during subsequent passages. The cultures showed consistent morphology and progressive expansion, forming homogeneous adherent monolayers.

In contrast, cells cultured in DMEM/F12 medium showed markedly reduced proliferative capacity after P1. Although initial attachment after reseeding was occasionally observed, further expansion of the cultures was limited, and stable growth could not be maintained.

Primary canine luteal cells cultured in RPMI 1640 medium retained stable morphology and proliferative capacity up to P3. Cells at P2 and P3 exhibited morphology comparable to that observed after P1, with a predominance of spindle-shaped cells and a minor population of polygonal cells. Cultures at P2 or P3 were used for further analyses and for cryopreservation.

#### 3.4.3. Expansion Kinetics and Population Doubling Time

Quantitative analysis of culture expansion revealed marked differences in growth kinetics between primary canine luteal cells maintained in RPMI 1640 and DMEM/F12 media (Table 2).

Cells cultured in RPMI 1640 medium reached passaging confluence substantially faster than cells maintained in DMEM/F12 medium. During the initial expansion stage (P0 → P1), RPMI 1640 cultures required 3.8 ± 0.4 days to reach confluence, whereas DMEM/F12 cultures required 9.2 ± 1.3 days. Following the first passage, RPMI 1640 cultures continued to exhibit rapid growth, reaching confluence within 5.0 ± 0.0 days at P1 → P2 and 4.8 ± 0.8 days at P2 → P3. In contrast, DMEM/F12 cultures required 13.2 ± 1.3 days to reach confluence after the first passage.

Consistent with these observations, population doubling times were considerably shorter in RPMI 1640 cultures than in DMEM/F12 cultures. The shortest doubling time was observed in RPMI 1640 cultures during the P0 → P1 expansion stage (37.0 ± 5.3 h), whereas DMEM/F12 cultures exhibited doubling times of 92.9 ± 12.7 h at P0 → P1 and 198.9 ± 15.6 h after the first passage. In RPMI 1640 medium, doubling times remained relatively stable during subsequent passages, ranging from 65.5 ± 9.4 h to 69.8 ± 6.0 h.

Despite the observed differences in proliferation kinetics, cell viability remained consistently high in both culture systems throughout expansion, ranging between 95% and 99%.

Overall, primary canine luteal cells maintained in RPMI 1640 medium demonstrated superior expansion capacity characterized by faster proliferation and shorter population doubling times compared with cells cultured in DMEM/F12 medium.

### 3.5. Immunofluorescence Characterization of Primary Canine Luteal Cells

**Figure 6:**
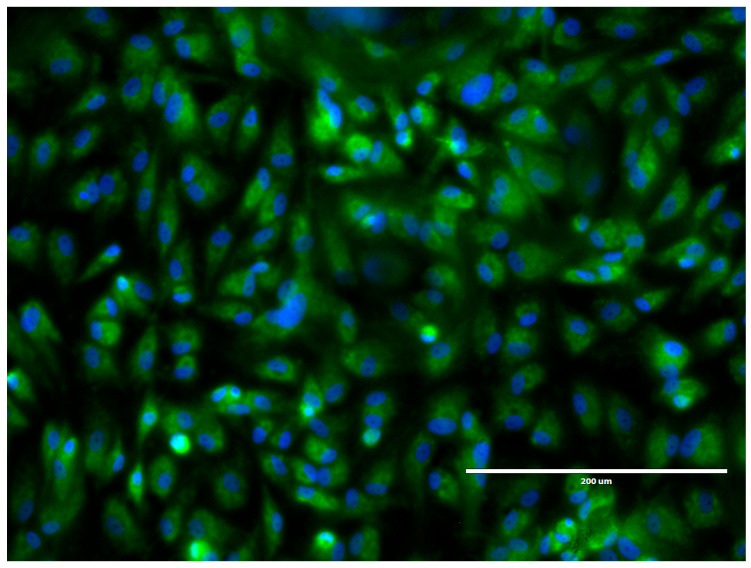

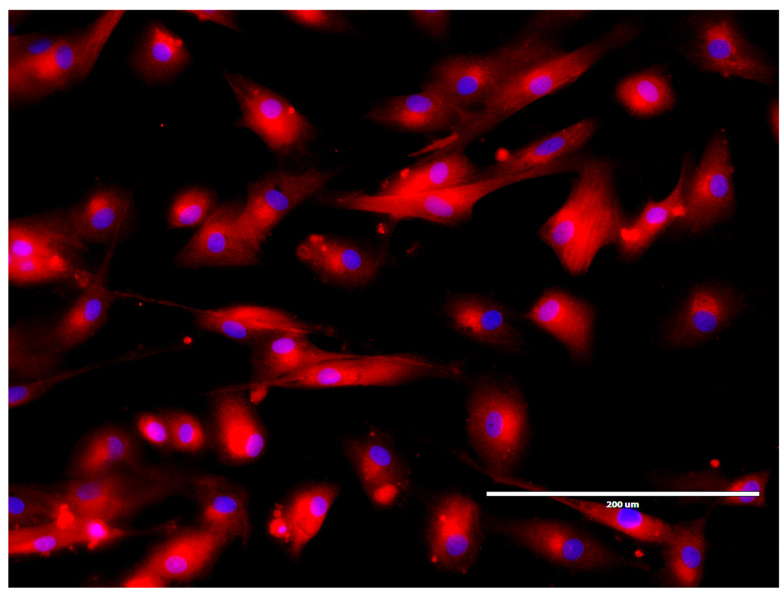
Representative immunofluorescence staining of primary canine luteal cell cultures demonstrating expression of steroidogenic markers. Nuclear staining with 4′,6-diamidino-2-phenylindole (DAPI) is shown in blue. Immunofluorescence detection of HSD3B1 is shown in green and STAR in red. Cells exhibit predominantly spindle-shaped morphology with a minor population of polygonal epithelial-like cells. Negative controls were performed by omitting the primary antibody and incubating cells with secondary antibody only. Additional representative stainings from all donor-derived cultures together with the corresponding negative controls are presented in Supplementary Figure S1.

Primary canine luteal cells were characterized by immunofluorescence staining for steroidogenic markers STAR and HSD3B1. Representative fluorescence images are shown in Figure 6. To assess staining reproducibility, immunofluorescence analyses were performed on cultures derived from all five donor animals.

Immunofluorescence analysis demonstrated clear cytoplasmic expression of STAR and HSD3B1 in cultured cells. STAR immunoreactivity was detected as a red fluorescence signal, while HSD3B1 expression was observed as a green fluorescence signal. Cell nuclei were visualized by DAPI staining (Table 3).

Strong cytoplasmic expression of both STAR and HSD3B1 was consistently observed in cultures derived from all donor animals and at all evaluated passage numbers. Virtually all cultured cells demonstrated strong cytoplasmic immunoreactivity for both markers, indicating a predominantly steroidogenic phenotype. Comparable staining patterns and signal intensity were observed in cultures maintained in DMEM/F12 after P1 as well as in RPMI 1640 cultures evaluated at P2 and P3, suggesting preservation of this phenotype during in vitro expansion. No specific fluorescence signal was detected in secondary-antibody-only negative controls. Representative images obtained from all donor-derived cultures together with the corresponding negative controls are presented in Supplementary Figure S1. Detailed immunofluorescence assessment across culture conditions and passages is summarized in Supplementary Table S2.

The fluorescence signal was predominantly localized in the cytoplasmic region surrounding the nuclei, consistent with the intracellular localization of steroidogenic enzymes.

Importantly, positive immunoreactivity for both markers was observed in cultures established using different basal media and isolation conditions. No qualitative differences in STAR or HSD3B1 expression were detected between cultures obtained using RPMI 1640 or DMEM/F12 medium.

The results confirmed that the isolated cells retained steroidogenic characteristics typical of functional luteal cells under in vitro culture conditions.

### 3.6. Functional Activity of Primary Canine Luteal Cells

The functional activity of primary canine luteal cells was evaluated by measuring P4 secretion under in vitro conditions. Cultured cells produced measurable amounts of P4, confirming the preservation of steroidogenic activity after isolation and during subsequent culture.

P4 secretion was observed in cultures established using different isolation conditions and basal media. No qualitative differences in hormone production were detected between cells cultured in RPMI 1640 and DMEM/F12 media, indicating that the steroidogenic capacity of the isolated luteal cells was maintained independently of the culture conditions applied.

In addition to basal P4 secretion, cultured cells responded to stimulation with forskolin, a known activator of the cAMP signaling pathway involved in steroidogenesis. Exposure to forskolin resulted in an increase in P4 production compared with untreated control cultures, further confirming the functional competence of the isolated luteal cells.

These results demonstrate that primary canine luteal cells retained both basal and stimulated steroidogenic activity in vitro, supporting their suitability as a functional model for studies of luteal physiology.

**Figure 7:**
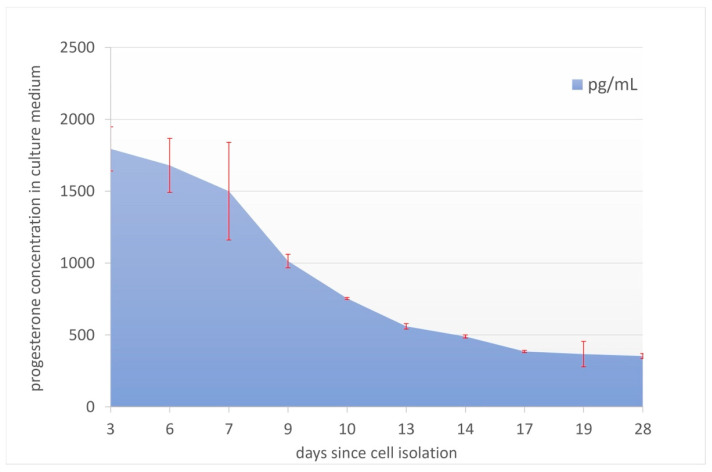
Time-dependent progesterone (P4) secretion by primary canine luteal cells cultured in vitro. P4 concentrations were measured in culture supernatants using a competitive ELISA assay at defined time points following cell isolation. Results are expressed as P4 concentration (pg/mL) and presented as mean ± SD (*n* = 5 independent cultures). P4 secretion was highest during the early stages of culture and gradually decreased over time, reaching a relatively constant basal level after approximately two weeks of culture. Statistical analysis demonstrated a significant effect of culture duration on progesterone secretion (Friedman test, χ^2^ = 42.51, df = 9, *p* < 0.0001).

A detailed quantitative analysis of time-dependent P4 secretion is provided in Section 3.8 and illustrated in Figure 7.

### 3.7. Cryobanking of Primary Canine Luteal Cells

Primary canine luteal cells obtained from all donor animals were successfully expanded in vitro and subsequently cryopreserved. Cells were maintained in either DMEM/F12 or RPMI 1640 medium without switching between media within individual cultures. Parallel cultures in both media were established for donors 3–5, whereas cultures derived from donors 1 and 2 were maintained exclusively in DMEM/F12.

Clear differences in proliferative capacity were observed depending on the basal culture medium. Cells cultured in DMEM/F12 demonstrated limited expansion potential after the first passage (P1), and further propagation after passaging was restricted. Consequently, these cultures were cryopreserved at early passage. In contrast, cells maintained in RPMI 1640 exhibited stable proliferation after passaging and were successfully expanded to later passages. Sequential expansion in T75 culture flasks enabled further amplification of cell numbers, and RPMI cultures were cryopreserved at passages P2 and P3.

Prior to cryopreservation, cell number and viability were determined using the NC-200™ automated cell counter. Cells were cryopreserved at a target density of approximately 5 × 10^5^ cells per cryovial (±20%), with the actual number of cells per cryovial ranging from approximately 4.6 × 10^5^ to 5.4 × 10^5^ cells.

In total, 39 cryovials were established, corresponding to approximately 1.95 × 10^7^ cells deposited in the cryobank. The average number of cells per cryovial was approximately 5.0 × 10^5^ cells. RPMI cultures consistently yielded higher and more reproducible cell numbers compared with DMEM/F12 cultures, allowing cryopreservation at multiple passages.

Detailed information regarding cryopreserved cell numbers and their distribution among donors, culture media, and passages is presented in Table 4 and Table 5.

### 3.8. Mycoplasma Screening

All analyzed primary canine luteal cell cultures tested negative for mycoplasma contamination using the MycoStrip™ assay. No evidence of mycoplasma contamination was detected at any evaluated stage of culture expansion, supporting the microbiological quality of the established cultures and cryopreserved cell stocks.

### 3.9. Progesterone Secretion by Primary Canine Luteal Cell Cultures

P4 secretion by primary canine luteal cell cultures was monitored at defined time points following cell isolation. P4 concentrations in culture supernatants were determined using a competitive ELISA assay and calculated from a standard curve as described in the Section 2.

Cell-free culture medium incubated under identical conditions and analyzed as a background control contained 103 ± 2.16 pg/mL progesterone. This value was substantially lower than the progesterone concentrations detected in luteal cell cultures throughout the study.

Primary luteal cells secreted measurable amounts of P4 throughout the entire observation period. The highest P4 concentrations were detected during the early stages of culture, reaching approximately 1794.6 ± 153.5 pg/mL on day 3 and 1679.6 ± 187.5 pg/mL on day 6 after isolation. Subsequently, P4 secretion gradually decreased over time, reaching 1500.1 ± 339.5 pg/mL on day 7 and 1014.6 ± 46.8 pg/mL on day 9.

A further decline in P4 production was observed during later stages of culture. P4 concentrations decreased to 753.9 ± 7.1 pg/mL on day 10 and continued to decline to 559.99 ± 18.9 pg/mL on day 13 and 489.23 ± 10.5 pg/mL on day 14. At prolonged culture times, P4 secretion stabilized at lower levels, reaching 384.9 ± 7.6 pg/mL on day 17, 367.1 ± 88.5 pg/mL on day 19, and 353.0 ± 17.2 pg/mL on day 28.

Overall, P4 secretion showed a progressive time-dependent decrease, with approximately a five-fold reduction between day 3 and day 28 of culture. Statistical analysis confirmed a significant effect of culture duration on progesterone secretion (Friedman test, χ^2^ = 42.51, df = 9, *p* < 0.0001).

The temporal changes in P4 secretion are presented in Figure 7.

The highest P4 concentrations were observed between day 3 and day 7, after which a marked reduction in hormone secretion occurred between day 7 and day 13. From approximately day 14 onward, P4 concentrations showed only minor fluctuations and remained within a relatively narrow range between approximately 353 and 489 pg/mL, indicating a plateau phase of P4 secretion during long-term culture. In particular, P4 concentrations measured on days 17, 19, and 28 remained comparable (384.9 ± 7.6 pg/mL, 367.1 ± 88.5 pg/mL, and 353.0 ± 17.2 pg/mL, respectively), suggesting that P4 secretion remained within a relatively narrow range after approximately two weeks of culture.

Despite the decline in hormone secretion over time, P4 remained detectable until the end of the observation period, indicating preservation of steroidogenic function throughout long-term culture.

### 3.10. Stimulation of Progesterone Secretion by Forskolin

The responsiveness of primary canine luteal cells to hormonal stimulation was evaluated using forskolin, an activator of adenylate cyclase and intracellular cAMP production. P4 secretion was measured in culture supernatants after incubation with increasing concentrations of forskolin as described in the Materials and Methods section (Section 2.12. Forskolin stimulation of progesterone secretion).

Forskolin stimulation resulted in a dose-dependent increase in P4 secretion compared with unstimulated control cultures. When normalized to basal secretion levels (E/E_0_), P4 production increased to 2.19 ± 0.19-fold at 1 μM, 2.40 ± 0.21-fold at 5 μM, and 2.72 ± 0.09-fold at 10 μM forskolin.

The strongest stimulation of P4 secretion was observed at 10 μM forskolin, corresponding to an approximately 2.7-fold increase compared with basal secretion levels. Even the lowest tested concentration (1 μM) induced a clear increase in P4 production, indicating high sensitivity of primary canine luteal cells to cAMP-mediated stimulation.

The observed dose-dependent response confirms that the cultured cells retained responsiveness to intracellular signaling pathways regulating P4 synthesis. These findings are consistent with the positive immunofluorescent staining for STAR and HSD3B1.

Statistical analysis demonstrated a significant effect of forskolin concentration on progesterone secretion (Friedman test, χ^2^ = 6.50, df = 2, *p* = 0.039).

**Figure 8:**
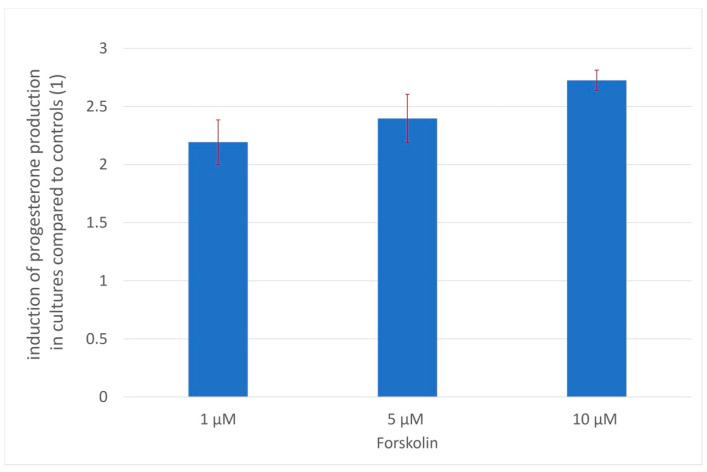
Effect of forskolin on progesterone (P4) secretion by primary canine luteal cells. P4 concentrations were determined using a competitive ELISA assay. Results are expressed as E/E_0_ (mean ± SD, *n* = 4), where E/E_0_ represents the fold increase in P4 secretion relative to untreated control cultures. Expression of results as E/E_0_ allowed comparison between independent cultures with different basal secretion levels. Statistical analysis demonstrated a significant effect of forskolin concentration on progesterone secretion (Friedman test, χ^2^ = 6.50, df = 2, *p* = 0.039).

The results of forskolin stimulation are presented in Figure 8 (E/E_0_ ratio).

## 4. Discussion

The present study establishes a reproducible and functionally validated in vitro model of the canine corpus luteum based on primary luteal cell cultures. Importantly, this work extends beyond a methodological description by demonstrating preserved endocrine function, including sustained progesterone secretion and responsiveness to regulatory stimulation. These findings support the use of canine luteal cells as a biologically relevant experimental system for investigating luteal physiology.

Consistent with previous reports, enzymatic isolation using collagenase yielded viable and adherent luteal cell populations capable of maintaining steroidogenic activity in vitro [39]. However, studies on canine luteal cells remain limited, and previously described models have largely focused on short-term cultures without extensive functional validation [39,48,49]. The present study addresses this gap by demonstrating sustained endocrine activity and reproducible culture performance across multiple donors.

A key finding of this study is the identification of culture conditions that support both functional maintenance and expansion of canine luteal cells. While DMEM/F12 medium allowed initial establishment of primary cultures, RPMI 1640 supplemented with fetal bovine serum provided superior support for cell proliferation, stability across passages, and reproducible expansion. Quantitative analysis of expansion kinetics demonstrated substantially shorter population doubling times and faster attainment of passaging confluence in RPMI 1640 cultures compared with DMEM/F12 cultures. These observations indicate that optimized culture conditions are critical for developing robust in vitro systems suitable for downstream applications.

The functional competence of the established cultures was confirmed by sustained progesterone secretion and preserved responsiveness to forskolin stimulation. Activation of the cyclic AMP pathway resulted in a marked increase in progesterone production, demonstrating that key intracellular regulatory mechanisms governing steroidogenesis remain intact in vitro. Together with the expression of steroidogenic markers, these findings confirm that the cultured cells retain essential features of luteal endocrine function and represent a functional model rather than merely a primary cell preparation.

The progressive decline in progesterone secretion observed during prolonged culture may reflect adaptation of primary luteal cells to in vitro conditions. Following isolation, cells are removed from the complex endocrine and paracrine environment of the corpus luteum and no longer receive physiological luteotrophic stimulation. In addition, the presence of tissue explants during early culture stages may have contributed to the initially higher progesterone concentrations. Despite this decline, cultured cells retained strong expression of STAR and HSD3B1 and remained responsive to forskolin stimulation after passaging, supporting the preservation of key steroidogenic characteristics during in vitro expansion. Therefore, the observed decline in progesterone production may reflect partial adaptation to in vitro conditions and reduced steroidogenic activity over time rather than complete loss of luteal phenotype.

Although mixed cellular populations are inherently associated with primary corpus luteum cultures, several findings argue against extensive overgrowth by non-steroidogenic stromal cells. Cultures maintained in RPMI 1640 retained proliferative capacity up to P3 while preserving strong STAR and HSD3B1 expression together with responsiveness to forskolin stimulation. In contrast, cultures maintained in DMEM/F12 displayed limited expansion after passaging. While culture medium alone cannot determine cellular identity, the observed growth characteristics, combined with the persistence of steroidogenic markers and functional activity, strongly support the predominance of steroidogenic luteal cells within the established cultures.

From a translational perspective, the established model provides a valuable platform for investigating regulatory mechanisms controlling luteal function, including luteolysis, endocrine signaling, and progesterone synthesis. This is particularly relevant in the domestic dog, in which the corpus luteum represents the sole source of progesterone during both diestrus and pregnancy [25,46,47]. Consequently, any disruption of luteal function may have direct implications for fertility and pregnancy maintenance. The availability of a controlled in vitro system enables detailed analysis of factors affecting steroidogenesis, including hormonal regulators, inflammatory mediators, and pharmacological agents.

An additional strength of this study is the successful expansion and cryopreservation of primary canine luteal cells, enabling the generation of standardized cell stocks for future applications. The establishment of a structured cryobank enhances reproducibility, reduces dependence on donor availability, and facilitates longitudinal and comparative studies. Furthermore, all analyzed cultures tested negative for mycoplasma contamination, supporting the microbiological quality of the established cultures and cryopreserved cell stocks.

A limitation of the present study is the relatively small number of donor animals, which reflects the limited availability of suitable samples in routine veterinary practice. Additionally, the use of mixed luteal cell populations may limit the ability to assess cell-type-specific responses. Although the available immunofluorescence and functional data strongly support the steroidogenic luteal identity of the established cultures, the presence of a minor population of non-steroidogenic ovarian cells cannot be completely excluded. Additional characterization using broader lineage-specific marker panels and flow cytometric analyses would further refine the phenotypic characterization of the model and should be considered in future studies.

The present study was not designed to compare biological properties between individual passages. Instead, passage number reflected the expansion potential of cultures maintained under different culture conditions. Nevertheless, steroidogenic marker expression and responsiveness to forskolin were preserved in post-passage cultures, suggesting maintenance of key steroidogenic characteristics during in vitro expansion. Future studies should investigate passage-dependent changes in steroidogenic activity, gene expression, and hormone production in greater detail.

Although the present study demonstrated preserved steroidogenic activity and cAMP responsiveness, additional functional characterization would further strengthen the biological relevance of the model. Future studies will focus on the evaluation of luteotrophic and luteolytic signaling pathways, including LH/hCG responsiveness, prostaglandin-mediated regulation, steroidogenic gene expression, and mechanisms associated with luteal regression. Such investigations will provide a more comprehensive understanding of the functional capabilities of the established canine luteal cell culture system.

## 5. Conclusions

In conclusion, this study establishes a reproducible and functionally validated primary canine luteal cell culture model suitable for in vitro investigations of corpus luteum biology. The cultured cells retained key steroidogenic characteristics, including expression of the steroidogenic markers STAR and HSD3B1, sustained progesterone secretion, and responsiveness to cAMP-mediated stimulation. Furthermore, optimized culture conditions enabled efficient expansion, successful cryopreservation, and recovery of viable cultures following thawing.

Although progesterone secretion gradually declined during prolonged culture, functional steroidogenic competence was retained after passaging, supporting the maintenance of essential luteal cell functions in vitro. While the presence of a minor population of non-steroidogenic ovarian cells cannot be completely excluded, the combined morphological, immunofluorescence, and functional findings strongly support the predominance of steroidogenic luteal cells within the established cultures.

Overall, this model provides a valuable platform for mechanistic and translational studies of luteal physiology, endocrine regulation, and progesterone-dependent reproductive processes in the domestic dog. Future studies should further refine the phenotypic characterization of the cultures and expand their functional validation through investigation of luteotrophic and luteolytic signaling pathways.

## Figures and Tables

**Table 1:** Details of isolation attempts and culture establishment efficiency for individual donors.

Donor	Number of Flasks	MSC Attachment Coating	Basal Medium	Explant Attachment	Cell Outgrowth	Stable Culture
1	6	4 Yes/2 No	DMEM/F12	Yes (coated only)	Yes (coated only)	Yes (coated only)
2	6	4 Yes/2 No	DMEM/F12	Yes (coated only)	Yes (coated only)	Yes (coated only)
3	6	Yes	3 DMEM/F12/3 RPMI	Yes	Yes	Yes
4	6	Yes	3 DMEM/F12/3 RPMI	Yes	Yes	Yes
5	6	Yes	3 DMEM/F12/3 RPMI	Yes	Yes	Yes

Notes: Uncoated flasks showed poor attachment of tissue fragments, and only single adherent cells were observed. Stable primary cultures were not established under these conditions.

**Table 2:** Quantitative comparison of expansion kinetics of primary canine luteal cells cultured in DMEM/F12 and RPMI 1640 media.

Medium	Expansion Stage	Time to Passage (Days)	Population Doubling Time (h)	Viability (%)
DMEM/F12	P0 → P1	9.2 ± 1.3	92.9 ± 12.7	95–99
DMEM/F12	P1	13.2 ± 1.3	198.9 ± 15.6	95–99
RPMI 1640	P0 → P1	3.8 ± 0.4	37.0 ± 5.3	95–99
RPMI 1640	P1 → P2	5.0 ± 0.0	69.8 ± 6.0	95–99
RPMI 1640	P2 → P3	4.8 ± 0.8	65.5 ± 9.4	95–99

**Notes:** Data are presented as mean ± SD from five independent donor-derived cultures. Population doubling time was calculated based on harvested and seeded cell numbers recorded at each passage.

**Table 3:** Immunofluorescence detection of steroidogenic markers in primary canine luteal cell cultures.

Marker	Fluorescence Color	Cellular Localization	Expression
STAR	Red	Cytoplasmic	Positive
HSD3B1	Green	Cytoplasmic	Positive
DAPI	Blue	Nuclear	Positive

**Table 4:** Distribution and quantitative characteristics of cryobanked luteal cells according to culture medium and passage number.

Medium	Passage	Number of Cryovials	Mean Cells/Vial	SD	Total Banked Cells
DMEM/F12	P1	15	5.03 × 10^5^	7.3 × 10^4^	7.54 × 10^6^
RPMI 1640	P2	12	4.79 × 10^5^	6.3 × 10^4^	5.75 × 10^6^
RPMI 1640	P3	12	5.20 × 10^5^	6.5 × 10^4^	6.24 × 10^6^
Total		39	5.01 × 10^5^	6.8 × 10^4^	1.95 × 10^7^

**Table 5:** Distribution of cryobanked luteal cells according to donor and culture medium.

Donor	Medium	Number of Cryovials	Total Banked Cells	Mean Cells/Vial
1	DMEM/F12	3	1.40 × 10^6^	4.68 × 10^5^
2	DMEM/F12	3	1.53 × 10^6^	5.10 × 10^5^
3	DMEM/F12	3	1.49 × 10^6^	4.96 × 10^5^
3	RPMI 1640	8	3.76 × 10^6^	4.70 × 10^5^
4	DMEM/F12	3	1.60 × 10^6^	5.33 × 10^5^
4	RPMI 1640	8	4.20 × 10^6^	5.25 × 10^5^
5	DMEM/F12	3	1.52 × 10^6^	5.07 × 10^5^
5	RPMI 1640	8	4.03 × 10^6^	5.04 × 10^5^

## Data Availability

All data generated or analyzed during this study are included in this published article and its Supplementary Information files. Additional data are available from the corresponding author on reasonable request.

## Supplementary Materials

The following supporting information can be downloaded at: https://www.mdpi.com/article/10.3390/biomedicines14071444/s1, Figure S1. Representative immunofluorescence staining of primary canine luteal cell cultures derived from five independent donor animals. Table S1: Individual characteristics of donor bitches. Table S2: Summary of immunofluorescence evaluation of steroidogenic markers in primary canine luteal cell cultures maintained under different culture conditions and passages.

## Abbreviations

The following abbreviations are used in this manuscript:

B_0_	maximum binding signal
BSA	bovine serum albumin
cAMP	cyclic adenosine monophosphate
CL	corpus luteum
CO_2_	carbon dioxide
DAPI	4′,6-diamidino-2-phenylindole
DMEM/F12	Dulbecco’s Modified Eagle Medium/Ham’s F-12 nutrient mixture
DMSO	dimethyl sulfoxide
E/E_0_	relative progesterone secretion (fold change vs control)
ELFA	enzyme-linked fluorescent assay
ELISA	enzyme-linked immunosorbent assay
FBS	fetal bovine serum
FFPE	formalin-fixed paraffin-embedded
FITC	fluorescein isothiocyanate
H&E	hematoxylin and eosin
HSD3B1	3β-hydroxysteroid dehydrogenase type 1
LLC	large luteal cells
MSC	mesenchymal stem cell (MSC Attachment Solution)
NGS	normal goat serum
NSB	non-specific binding
OHE	ovariohysterectomy
P4	progesterone
PBS	phosphate-buffered saline
PBST	phosphate-buffered saline with Tween-20
P0–P3	cell culture passages (passage 0–3)
RPMI 1640	Roswell Park Memorial Institute medium 1640
SD	standard deviation
SLC	small luteal cells
STAR	steroidogenic acute regulatory protein
