# Supplementary material for: Functional In Vitro Model of the Canine Corpus Luteum: Isolation, Culture and Characterization of Steroidogenically Active Luteal Cells
Source: Biomedicines. 2026 Jun 25;14(7):1444. doi: 10.3390/biomedicines14071444 (PMC13405799; doi:10.3390/biomedicines14071444)
Supplement: Supplementary file 1 [file biomedicines-14-01444-s001.zip › biomedicines-4342138- supplementary FigureS1.pdf]

Supplementary Figure S1

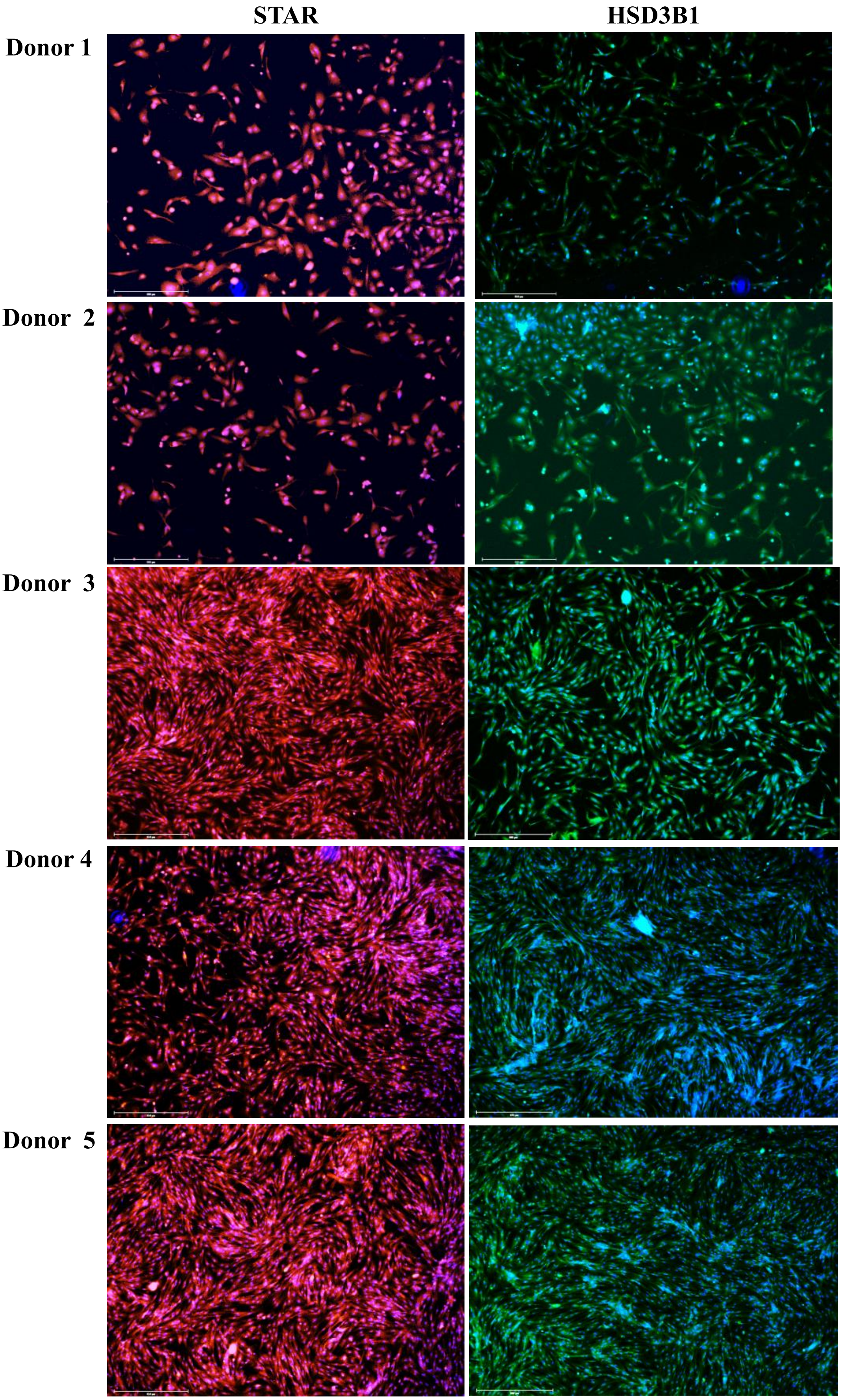

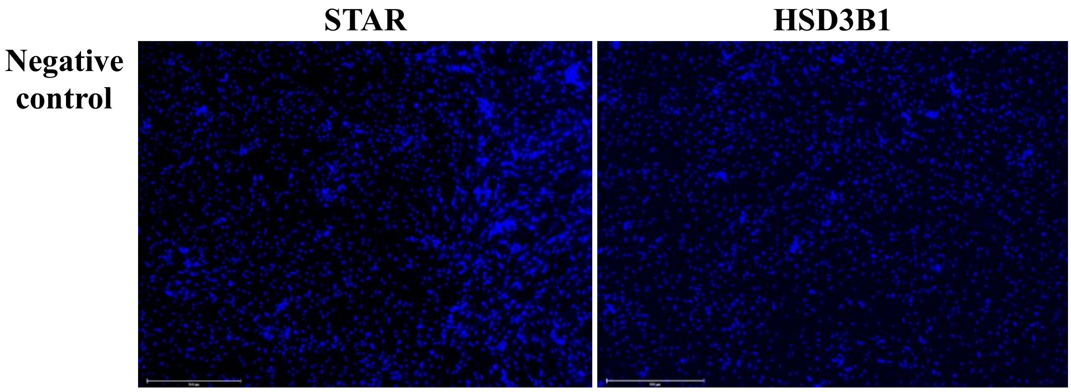

Figure S1. Representative immunofluorescence staining of primary canine luteal cell cultures derived from five independent donor animals. The left column shows STAR staining, and the right column shows HSD3B1 staining. Strong positive staining was observed in all donor-derived cultures, whereas no specific fluorescence signal was detected in the secondary-antibody-only negative controls.
