# Supplementary material for: Functional In Vitro Model of the Canine Corpus Luteum: Isolation, Culture and Characterization of Steroidogenically Active Luteal Cells
Source: Biomedicines. 2026 Jun 25;14(7):1444. doi: 10.3390/biomedicines14071444 (PMC13405799; doi:10.3390/biomedicines14071444)
Supplement: Supplementary file 1 [file biomedicines-14-01444-s001.zip › biomedicines-4342138- supplementary Table S1.pdf]

Supplementary Table S1

Dog ID	Breed	Age (years)	Onset of estrus (first day of clinical signs)	Progesterone concentration (ng/mL)	Date of P4 measurement	Date of OHE	Interval P4–OHE (days)	Preoperative blood tests (hematology and biochemistry)	Clinical status
1	German Shepherd Dog	3	06.10.2024	48,42	21.10.2024	29.10.2024	8	Within reference ranges or minor clinically insignificant deviations	Clinically healthy
2	Beagle	9	02.11.2024	47,19	19.11.2024	25.11.2024	6	Not available	Clinically healthy
3	Shiba Inu	4	18.10.2024	42,64	04.11.2024	02.12.2024	28	Within reference ranges or minor clinically insignificant deviations	Clinically healthy
4	Shiba Inu	5	27.10.2025	72,18	12.11.2025	12.11.2024	0	Within reference ranges or minor clinically insignificant deviations	Clinically healthy
5	Chinese Crested Dog	6	24.12.2025	37,31	07.01.2026	19.01.2026	12	Within reference ranges or minor clinically insignificant deviations	Clinically healthy

Table S1. Individual characteristics of donor bitches.
