# Supplementary material for: Functional In Vitro Model of the Canine Corpus Luteum: Isolation, Culture and Characterization of Steroidogenically Active Luteal Cells
Source: Biomedicines. 2026 Jun 25;14(7):1444. doi: 10.3390/biomedicines14071444 (PMC13405799; doi:10.3390/biomedicines14071444)
Supplement: Supplementary file 1 [file biomedicines-14-01444-s001.zip › biomedicines-4342138- supplementary Table S2.pdf]

Supplementary Table S2

Culture medium	Passage	STAR expression	HSD3B1 expression	Signal intensity	Interpretation
DMEM/F12	P1	Positive	Positive	Strong (+++)	Steroidogenic phenotype maintained
RPMI 1640	P2	Positive	Positive	Strong (+++)	Steroidogenic phenotype maintained
RPMI 1640	P3	Positive	Positive	Strong (+++)	Steroidogenic phenotype maintained

Table S2. Summary of immunofluorescence evaluation of steroidogenic markers in primary canine luteal cell cultures maintained under different culture conditions and passages. Qualitative assessment based on immunofluorescence microscopy. Strong cytoplasmic immunoreactivity for both STAR and HSD3B1 was consistently observed in virtually all examined cells regardless of culture medium or passage number.
